# Quality of Life in Pediatrics With Intractable Epilepsy at King Abdulaziz Medical City, Jeddah, Saudi Arabia: A Cross-Sectional Study

**DOI:** 10.7759/cureus.42417

**Published:** 2023-07-25

**Authors:** Yasir O Marghalani, Ammar Aljabri, Abdulrahman H Kaneetah, Sultan G Alzahrani, Mohammed Hmoud, Ahmed Attar

**Affiliations:** 1 College of Medicine, King Saud Bin Abdulaziz University for Health Sciences College of Medicine, Jeddah, SAU; 2 College of Medicine, King Abdullah International Medical Research Center, Jeddah, SAU; 3 College of Medicine, University of Bisha, Bisha, SAU; 4 Department of Medicine, King Abdullah International Medical Research Center, Jeddah, SAU; 5 Department of Neuroscience, Ministry of the National Guard-Health Affairs, Jeddah, SAU; 6 College of Medicine, King Saud Bin Abdulaziz University for Health Sciences, Jeddah, SAU; 7 Department of Medicine, Hamilton Health Sciences Centre, Jeddah, SAU; 8 Department of Medicine, McMaster University, Hamilton, CAN

**Keywords:** quality of life, suicidal ideation, low to middle income countries, anti-epileptic drugs, person with epilepsy

## Abstract

Objective

The aim of this study was to assess the cognitive, emotional, social, and physical domains of quality of life (QoL) in pediatric patients with intractable epilepsy with an emphasis on depressed mood and suicidal ideation (SI).

Methods

This is a cross-sectional study conducted in pediatric neurology outpatient clinics in King Abdulaziz Medical City, Jeddah, Saudi Arabia. The sample consisted of 59 parents whose children aged 4‐14 years of either sex had intractable epilepsy. The Quality of Life in Childhood Epilepsy Questionnaire - 55 (QOLCE‐55) scale examined four domains of life: cognitive, emotional, social, and physical. Depressed mood and SI were part of the emotional domain.

Results

The mean ± SD age of children was 8.2 ± 3.25. The mean ± SD of overall QoL was 43.02 ± 15.70, which reflected a poor QoL. Age was not related to the QoL. Female gender was significantly associated with a lower overall QoL (P = 0.0477). Patients with comorbidities had statistically insignificant lower QoL in the cognitive, social, and physical domains in addition to lower overall QoL. Seven of nine participants who reported feeling down reported having SI in the last four weeks (P < 0.001).

Conclusions

An intractable epilepsy-imposed burden negatively impacts all domains of QoL. Furthermore, females experience lower overall QoL compared to males. Children with comorbidities also tend to have lower QoL scores, although the differences were statistically insignificant. Additionally, a history of feeling down is associated with SI.

## Introduction

Epilepsy is defined as experiencing one seizure with a more than 60% chance of developing another seizure within 10 years, or if reflex epilepsy is present [1]. Seizures are categorized as focal or generalized, depending on whether they involve a single cerebral hemisphere or both [2]. Typically, seizures tend to occur most frequently during childhood or after the age of 60 years [2]. With an annual incidence of 50 to 70 new cases per 100,000 population, epilepsy is considered a common disease [2]. However, there are limited epidemiological data available on the prevalence and incidence of epilepsy in Saudi Arabia [3,4]. Nonetheless, it has been reported that the prevalence of epilepsy in Saudi Arabia is 6.5 per 1000 people overall and 2.5 per 1000 in pediatric cases [5]. The morbidity caused by epilepsy is significant not only to the patient but also to all members of the family; moreover, it is the most common illness referred to pediatric neurological clinics [6].

Fortunately, approximately 70% of seizure cases in epilepsy can be effectively managed using antiepileptic drugs (AEDs). However, the remaining 30% of cases are referred to as intractable epilepsy, which means that despite undergoing two or more trials of AEDs with appropriate choices and doses, the seizures remain uncontrolled [4]. Intractable epilepsy poses a greater socioeconomic burden, as even with long-term treatment and optimal use of AEDs, around 30% of patients will develop this condition [5]. Epilepsy is a progressive and complex disorder that has unpredictable and debilitating effects on the quality of life (QoL). Additionally, individuals with epilepsy often face social stigma and experience a significant economic burden, which affects both the affected child and their family members [7,8].

Families with children who have epilepsy are more likely to encounter social and marital issues, dysfunctional parent-child relationships, and increased levels of stress, depression, and anxiety compared to families without epilepsy, making this an important area for investigation [9]. Furthermore, a child's neurocognitive impairment serves as the strongest predictor of maternal clinical depression, and the poor psychological and mental well-being of mothers is associated with adverse health outcomes and psychopathology in their children [10, 11].

Intractable epilepsy is a universally burdensome disease that impacts various aspects of an individual's personal and family QoL, particularly in low- to middle-income countries. However, there is a lack of evidence regarding these effects, especially in the Middle East in general and Saudi Arabia specifically. Another cross-sectional study conducted in the central region of Saudi Arabia, which aimed to explore similar objectives as ours, concluded that intractable epilepsy significantly affects QoL [12]. Based on this observation, we investigated whether there was an overall interregional difference between the central and western regions of Saudi Arabia. Additionally, considering the importance and scarcity of literature on the bi-directional effects between personal and family QoL and outcomes of intractable epilepsy in pediatric cases [8,9,10,11], we formulated a hypothesis for our study. The aim of our study was to assess the QoL in children with intractable epilepsy using the Quality of Life in Childhood Epilepsy - 55 (QOLCE-55) scale [13] at King Abdulaziz Medical City, Jeddah (KAMC-J), Saudi Arabia, in 2021.

## Materials and methods

This research was a cross-sectional study conducted at KAMC-J, Jeddah, Saudi Arabia, using a self-administered questionnaire that was filled out by caregivers of patients with epilepsy visiting outpatient neurology clinics. 

Using convenience availability sampling, we determined the sample size based on the number of caregivers of children with intractable epilepsy visiting the outpatient neurology clinics at KAMC-J. A similar study was published in Riyadh in 2020, which assessed the QoL of 59 pediatric patients with intractable epilepsy in a large pediatric university hospital. We used this as a reference for determining the minimal sample size [12]. However, when attempting to calculate the sample size using Raosoft.com sample size calculator, using the Centers for Disease Control's (CDC) nationwide prevalence of 0.6% for epilepsy, the estimated sample size was 10 with a 5% margin of error and a 95% confidence interval, which was deemed inadequate to yield statistically significant results.

It appears that the available sample size for our study may be limited due to the scarcity of caregivers of children with intractable epilepsy visiting the outpatient neurology clinics at KAMC-J. This limitation may affect the statistical power and generalizability of the study findings.

Inclusion criteria consisted of the pediatric age group excluding neonates and infants, which is from 4 to 17 years, and satisfying the definition of intractable epilepsy, which is inadequate control of symptoms despite using three AEDs of optimal dosage and choice. Both males and females were included in the study. The exclusion criteria comprised adequate control of symptoms. 

This questionnaire is composed of a demographic section, which gathers information about epileptic patients like age (in years), gender, and presence of comorbidities. As for QoL, Quality of Life in Childhood Epilepsy (QOLCE-55) was used as it is a validated instrument that has been translated, validated, and previously used in a similar setting. It is recommended by The National Institute of Neurological Disorders and Stroke Common Data Elements for assessing the QoL in patients with epilepsy. Parents must answer 55 questions regarding how often their children experience certain problems compared to other children of their age during the past four weeks. The QOLCE-55 section includes the following domains: cognitive, emotional, social, and physical functioning. Each item is on a 6-point Likert scale and includes anchors that are subjectively rated based on perceived QoL (e.g., 1 = very often, 2 = fairly often, 3 = sometimes, 4 = almost never, 5 = never, 6 = non-applicable). Answers are translated into a 0- to 100-point scale (1 = 0, 2 = 25, 3 = 50, 4 = 75, 5 = 100). Scores are composed of averages for each of the four domains, and an overall QoL score is derived by summing all the individual scores. Higher scores reflected a better QoL. Patients who score 75 are considered to have a good QoL. High internal consistency has been found for the QOLCE. QOLCE-55 was translated into Arabic by native Arabic speakers. English and Arabic versions of QOLCE-55 are available upon request as a supporting file: NRJ21J-253-10 Approved Questionnaire 1.pdf [12]. To clarify the missing responses, the questionnaire gives participants the freedom to dismiss questions according to their preferences. 

Data were entered and analyzed using JMP (SAS Institute, Kuala Lumpur, Malaysia). Categorical variables are presented as frequencies (percentages). Numerical data are described as mean ± SD. Independent sample t-test was used to determine whether there is a statistically significant difference between the means in two unrelated groups, and ANOVA was used to compare more than two means. For testing the association of the scores with age, linear regression was used. Any test was declared significant at a P-value of <0.05.

## Results

This study included 59 caregivers of Saudi children with epilepsy aged between 5 and 14 years, with a mean age of 8.2 ± 3.25 years and a slightly higher representation of females (55.9%). The most common comorbidity was global developmental delay (n = 28, 52%), followed by encephalopathy (n = 16, 29.6%), and spasticity (n = 5, 9%). Only two (3.63%) cases had attention deficit hyperactivity disorder (ADHD) and one (2%) had contractures. All baseline characteristics are summarized in Table 1.

**Table 1:** Baseline characteristics of patients (n = 59) SD: standard deviation, GDD: global developmental delay, ADHD: attention deficit hyperactivity disorder.

Variable	Frequency	Percentage
Age (mean ± SD)	8.2 ± 3.25	
Female	33	55.9
Male	26	44.1
GDD	28	52
Encephalopathy	16	29.6
Spasticity	5	9
ADHD	2	3.63
Contractures	1	2

The four domains of QoL were assessed: cognitive functioning with a mean score of 39.77 ± 23.8, emotional functioning with a mean score of 51.66 ± 17.68, social functioning with a mean score of 49.39 ± 23.65, and physical functioning with a mean score of 32.56 ± 21.74. The overall mean QoL score was 43.02 ± 15.70, which was low and reflected the negative impact of intractable epilepsy. The mean scores for all QoL domains are summarized in Table 2.

**Table 2:** Mean of overall and separate domains of QoL (n = 59)

Domain	Mean	Standard Deviation
Overall QoL	43.02	15.7
Mean cognitive	39.77	23.8
Mean emotional	51.66	17.68
Mean E 2.1 A-K	54.47	17.65
Mean E 2.2 a-f	50.5	19.71
Mean social	49.39	23.65
Mean physical	32.56	21.74

**Figure 1:**
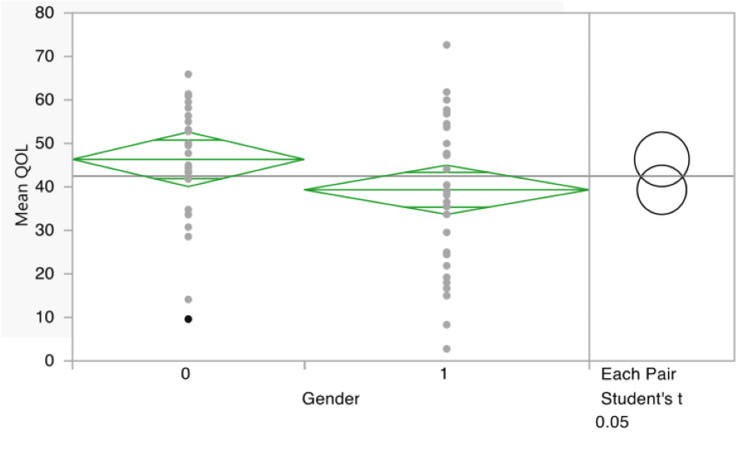
Mean overall quality of life scores between genders 0: male; 1: female.

Regarding gender differences, the only significant difference was the mean of the overall QoL (males = 46.35 ± 14.28, females = 39.34 ± 17.20; P = 0.0477) (Table 3 and Figure 1). Another significant difference in means was between the patients with global developmental delay (GDD) with a score of 57.93 ± 21.91 compared to patients without GDD with a score of 46 ± 16.91 in the emotional QoL second section. In addition, patients with spasticity had a mean emotional QoL of 66.78 ± 12.5 compared to 50.22 ± 18.42 in patients without spasticity. The mean QoL domains and the presence of certain comorbidity are shown in Table 4. For patients with spasticity (n = 5), the answers to the emotional domain, item 2 could not have been disclosed by the participants.

**Table 3:** Differences in means between male and female patients

Gender	Mean QoL	Mean Cognitive	Mean Emotional	Mean Social	Mean Physical
Male (n = 26)	46.35 ± 14.28	51.5 ± 14.12	51.5 ± 14.12	50.69 ± 21.88	32.79 ± 19.93
Female (n = 33)	39.34 ± 17.20	51.43 ± 20.8	51.43 ± 20.8	48.28 ± 26.37	31.00 ± 21.80
P-value	0.0477*	>0.05	>0.05	>0.05	>0.05

**Table 4:** Relation of comorbidities with QoL domains SD: standard deviation, GDD: global developmental delay, ADHD: attention deficit hyperactivity disorder, NA: as participants answered, it is not available or applicable. *Statistically significant.

Variable	Overall QOL	Cognitive	Emotional	Emotional Items 1	Emotional Items 2	Social	Physical
No comorbidities (n = 19)	44.5 ± 13.3	39.5 ± 17.82	57 ± 11.34	58.6 ± 13.05	49.77 ± 14.75	52.18 ± 20.14	48.3 ± 22.37
No GDD (n = 25)	42.8 ± 13.25	37 ± 20.92	50.08 ± 16.67	54.33 ± 17.65	46 ± 16.91*	48.85 ± 22.35	32.25 ± 21.82
GDD (n = 28)	42.66 ± 18.3	40.56 ± 27.46	53.21 ± 20.21	53.16 ± 18.62	57.93 ± 21.91*	45.75 ± 23.86	36.08 ± 19.41
No encephalopathy (n = 38)	42.88 ± 16.5	39.61 ± 25.27	54.32 ± 16.37	55.50 ± 15.72	53.06 ± 19.36	46.82 ± 23.26	35.88 ± 21.56
Encephalopathy (n = 15)	42.37 ± 15.1	36.97 ± 22.69	45.85 ± 22.76	49.5 ± 22.54	49.04 ± 23.15	48.24 ± 23.03	29.93 ± 17.55
No spasticity (n = 49)	43.51±15.83	40.3 ± 24.4	50.22 ± 18.42*	52.48 ± 17.98	50.53 ± 19.73	48.59 ± 23	34.54 ± 20.6
Spasticity (n = 5)	38.2 ± 18.1	30.91 ± 26.12	66.78 ± 12.5*	62.73 ± 16.9	69.79 ± 17.14	37.14 ± 20.92	24.03 ± 23.34
No contractures (n = 48)	45.027 ± 14.7	43 ± 22.27	49.79	51.73 ± 17.18	50.97 ± 19.5	49.18 ± 21.16	35.89 ± 20.54
Contractures (n = 1)	15	0	75	75	NA	0	31.25
No ADHD (n = 52)	43.72 ± 15.68	40.46 ± 24.24	52.58 ± 18.11	53.94 ± 17.78	53.52 ± 19.13	47.46 ± 23.3	33.67 ± 21.24
ADHD (n = 2)	24.46 ± 14.6	12.17 ± 13.99	29.05 ± 19.72	39.34 ± 24.71	18.75 ± 14.73	50 ± 0	30.73 ± 3.682

The distribution of answers to item 2.1c (wished death during the last four weeks in the emotional domain) showed that three (6%) participants reported “very often” and six (13%) reported “fairly often.” Moreover, in Table 5, seven (77.78%) of the nine participants who reported suicidal ideation (SI) reported feeling down with a significant association (P < 0.001). Ten participants could not disclose whether their dependant felt down or had SI.

**Table 5:** Relation between feeling down and suicidal ideation in the last four weeks The difference between reported responses and the number of participants is due to the participants' preference not to answer.

Count (%)	Suicidal Ideation: No	Suicidal Ideation: Yes	Total
Feeling Down: No	36 (90)	2 (22.22)	38
Feeling Down: Yes	4 (10)	7 (77.78)	11
Total	40	9	49

## Discussion

The burden of epilepsy worldwide ranges from 4 to 10 cases per 1000 people, affecting approximately 50 million patients. In the Middle East region, a regional median prevalence rate of 2.3 has been reported. Locally, the prevalence of epilepsy is 6.54 per 1000 individuals, with a higher incidence of diagnosis occurring during childhood [14].

There is a consensus among researchers that children facing epilepsy generally experience a lower QoL compared to their peers [15, 16]. Moreover, intractable epilepsy imposes an even greater burden on patients, particularly in the physical and psychological domains [15, 16]. As the severity and frequency of seizures increase, patients become more vulnerable to depression and suicidal tendencies [17, 18]. In fact, specialized epilepsy centers have reported depression in up to half of their patients [17]. Additionally, Jacob et al. found that 60% of epilepsy patients experienced a sense of internal stigma, which could contribute to the development of psychopathology [19].

The current study aimed to observe the overall QoL scores, the cognitive, emotional, social, and physical components specifically, and report any associations with the patient’s demographics.

Regarding age, the results showed that there was no significant association with QoL as a whole or any of the individual domains. Similarly, Nagarathnam et al. [20] and Altwaijri et al. [12] found no significant association between age groups and overall QoL. On the contrary, Taylor et al. showed that as age increased, only scores related to self-esteem decreased, as adolescent patients experience difficulty forming their identity without feeling ashamed or stigmatized by the disease; however, 84% of our research participants were younger than 11.5 years, whereas Taylor et al. reported this finding in the 12-15-year age group [21]. Moreover, Shetty et al. [22] and Ohaeri et al. [23] showed more significant associations when older age groups had lower scores of physical pain, emotional health, and memory and language QoL; however, they observed this finding in an adult population unlike our pediatric participants, which may support the hypothesis that being burdened by the disease and its stigma for a longer period and exhaustion of caretakers leads to a decline in several domains in QoL.

When comparing the means of QoL between the two genders, the only statistically significant domain was the overall mean QoL, where females had a lower mean score (Figure 1 and Table 3). In harmony with our results, Haider et al. [24] and Buck et al. [25] demonstrated that females with epilepsy in Pakistan and multiple countries in Europe had a lower mean score of overall QoL; moreover, some of the postulated theories behind this finding were that female patients are more affected by feelings and worry regarding the mental and physical effects of AEDs in addition to a decreased feeling of support and social isolation when compared to male participants. To support this observation further, another study in Riyadh, Saudi Arabia, demonstrated that female pediatric patients with intractable epilepsy had a lower mean QoL both overall and in the social domain specifically [12]. Moreover, in another study in Ethiopia, this finding was further demonstrated in female patients with epilepsy having decreased attendance at school [26]. However, Nabukenya et al. [27] reported that females had a better health-related QoL; however, they reported its positive effect was observed in patients with a moderate frequency of seizures, which varies from our sample of patients who have intractable epilepsy. A study of newly diagnosed pediatric and adolescent epileptic patients found no significant association between females and males; however, they justified this discrepancy by pointing out that their population consisted of newly diagnosed adolescents [21].

Features of heavier disease burden such as encephalopathy, spasticity, contractures, and ADHD were associated with lower QoL scores across all domains except the emotional and social domains (Table 4). However, the lower scores did not reach statistical significance. In line with our results, Haider et al. [24] reported that patients with comorbidities and especially developmental delay had the poorest QoL. Moreover, Miller et al. claimed that having comorbidities was the best predictor for a poor QoL [28].

Screening for SI in this study revealed that nine (19%) of our participants reported a greater frequency during the last four weeks (Table 5). Moreover, we found that feeling low had a significant association with SI. Kanner et al. hypothesized that iatrogenic causes, among others, from AEDs contributed to this increased risk [29]. Furthermore, they reported the prevalence rates of SI among PWE were 25%, which is two to three times higher than that of the general population. Furthermore, PWE cases with concomitant psychological disease were 12 to 32 times more at risk [29]. In addition, Tian et al. [30] reported that the estimated annual suicide rate among PWE in the United States was 22% higher than that of the general population (16.89/100,000).

One of the limitations of the present study was the convenience sampling technique, which can lead to sampling bias. Moreover, the study was conducted in one hospital and therefore lacks clear generalizability. Unfortunately, the reliability and the psychiatric condition of the caregivers were not assessed. Another limitation is the response bias due to the survey‐based nature of the study. One strength is that this study focuses on a minor presentation of epilepsy in a region where literature is scarce. Moreover, this study uses a translated and validated questionnaire. 

With the data presented, we aspire to educate healthcare providers specifically and the general population generally about how detrimental the effects of intractable epilepsy are on the pediatric population's QoL.

## Conclusions

Epilepsy and intractable epilepsy burden patients’ QoL across multiple domains. Female patients have been found to have a lower QoL compared to male patients. Comorbidities were associated with lower overall QoL of life but were statistically insignificant. In addition, a history of feeling low was associated with SI. We recommend thoroughly educating both patients and their caregivers about epilepsy and easing their worries in addition to raising awareness in the public. Moreover, we recommend screening both patients and caregivers, who may be affected as well, for depression and suicidal ideation. We also recommend performing a multi-centered study surveying both the patients and their caregivers.
